# Incidence of Injuries, Illness and Related Risk Factors in Cross-Country Marathon Mountain Biking Events: A Systematic Search and Review

**DOI:** 10.1186/s40798-021-00357-z

**Published:** 2021-09-25

**Authors:** Kim Buchholtz, Mike Lambert, Lieselotte Corten, Theresa L. Burgess

**Affiliations:** 1 Division of Exercise Science and Sports Medicine, Department of Human Biology, University of Cape Town, Cape Town, South Africa; 2 Department of Physiotherapy, LUNEX International University of Health, Exercise and Sports, Differdange, Luxembourg; 3 School of Health Sciences (Physiotherapy), University of Brighton, Eastbourne, UK; 4 Division of Physiotherapy, Groote Schuur Hospital, University of Cape Town, Cape Town, South Africa; 5 Centre for Medical Ethics and Law, Faculty of Medicine and Health Sciences, Stellenbosch University, Cape Town, South Africa

**Keywords:** Mountain biking, Injury, Illness, Accidents, Cycling

## Abstract

**Background:**

Cycling is a popular global sport and method of transportation and a significant contributor to admissions to hospital emergency units following an injury. Mountain biking events present additional challenges with remote venues and isolated courses, for which on-site medical care is often provided, for both injury and illness occurring during races. National health data may not represent these unique events, and specific data on incidence of injury and illness in mountain biking events are essential. Therefore, the aim of this study was to review the available injury and illness literature, reporting methods and risk factors in cross-country mountain biking.

**Methods:**

Search engines PubMed, Scopus, CINAHL (EBSCOhost), Scopus, PEDro and the Cochrane Library were systematically searched, and a grey literature search was performed. Narrative analyses of the types, severity and area of injuries and illness type and severity were performed as pooling of data was impossible due to insufficient high-quality studies with the same injury and illness definitions.

**Results:**

Seven studies comprising 28,021 participants were included for analysis. Four to 71% of participants sustained an injury in a cross-country mountain bike event. Injuries to the skin were the most common, followed by bony injuries and concussion. Five to 47% of cyclists reported the onset of gastrointestinal symptoms post-event. The prevalence of illness during events ranged from 0.5 to 23.0%.

**Conclusion:**

The injury and illness definitions were varied and prevented clear comparisons between studies. Injury and illness present a concern in cross-country marathon mountain biking and should be investigated further to provide the true burden of these during race events.

*Registration*: This protocol has been registered with PROSPERO International prospective register of systematic reviews (No: CRD42019134586).

## Key Points

- Illness and injury present significant concern in cross-country marathon mountain biking for both elite and amateur cyclists.
- The true burden of these injuries and illnesses is difficult to determine due to varied reporting methods and definitions.
- Further research in injury and illness epidemiology, using standardised and current definitions, is required in cross-country marathon mountain biking.

## Background

Cycling is an increasingly popular sport and is used as both a recreational activity and a form of transport globally [1]. Cycling is made up of two main categories, ‘road’ (or street) and ‘mountain’ (or off-road) biking. Mountain bike racing is further described by the Union Cycliste Internationale (UCI) as cross-country Olympic, cross-country marathon, cross-country eliminator, downhill, four-cross, Enduro and Alpine Snow Bike [2]. Cross-country marathon includes both amateur and professional riders in the same races on the same routes, which are usually between 60 and 120 km and is the most common format for recreational cyclists in South Africa, based on the types of mountain biking events available [2]. Mountain biking includes highly technical riding through rough terrain, forest tracks, gravel pathways and steep downhills, with only a small percentage of riding on tarred roads [2]. Mountain bikes, while varying widely in design, have wider tyres with greater grip and suspension on either the front wheel, or both, in comparison with road bicycles [3]. Competitive races are becoming more demanding and have evolved into multi-stage races in both road and mountain cycling categories [4].

As participation in cycling has increased, conflicting evidence on the incidence of injury has emerged [3, 5]. In the USA, cycling injuries account for 13% of all exercise-related injuries reporting to emergency departments [5]. Ten percent of these injuries occurred during ‘sporting activity’ without classification into road or mountain biking [5]. Fifty percent of mountain bikers have reported at least one serious acute injury related to mountain biking and in professional mountain bikers this number increases to 80% [3]. The incidence of injury among cross-country marathon riders is 7.5 and 3.1 injuries per 1000 h in males and females, respectively [6].

Most data on cycling injuries report cases presenting to emergency departments. These data include both commuter and recreational/sports cyclists, and rarely differentiate the cycling categories in their records [7]. Many race events have onsite medical care, due to the remote locations, and riders who suffer injuries may not be admitted to hospital [8]. It follows that national cycling injury data may underestimate injuries at cycling race events because the less serious injuries in mountain bikers which occur at organised events may not need referral to hospital [7]. Also, the mechanisms, incidence and management of the injuries may differ between commuting and events. Therefore, epidemiological data of injuries during cycling race events are essential. Injuries in mountain biking events present a unique challenge to event organisers. Riders may present with a combination of muscle strains, joint injury, overuse injury and trauma related to falling, and there may be a lack of access to injured athletes related to the environment/terrain [9].

Illness in mountain biking is poorly reported. Most of the data are from a limited number of race events [10, 11]. Gastrointestinal illness, allergies, respiratory illness, dehydration, headaches and skin irritations are the most commonly reported illnesses in cycling events [10, 12, 13]. In the 2016 Olympic Games, approximately 5% of mountain bikers were treated for a variety of illnesses by their medical teams [10].

The reporting of injury and illness in events varies depending on the definitions used by the researchers. The International Olympic Committee uses a medical attention definition for both injury and illness, and includes all occurrences of injury or illness reported to the medical teams regardless of the effect on the athlete’s ability to continue training or to compete [10]. Severity of the injury or illness is determined by the number of days absent from training or competition, and more than one week is defined as ‘severe’ [10]. The Consensus Statement of Epidemiological Studies in Athletics defines injury as a ‘physical complaint or observable damage to the body produced by a transfer of energy of the athlete’ [11]. Illness is defined as a ‘physical or psychological complaint or manifestation by an athlete not related to injury’ [11]. When only including medical attention injuries and illness, it is possible to underestimate the incidence, as cyclists who are able to continue without medical intervention, or are self-treating would not be considered injured [7].

Injury and illness prevention programmes require knowledge of the aetiology and magnitude of the injury or illness problem within the context of the sport [14, 15]. The current available knowledge in cycling is largely based on commuter cycling and hospital admissions, rather than on site at race events. In this systematic review, we examined studies of injury and illness of competitors in cross-country marathon mountain biking events of different lengths with the aim of providing a comprehensive review of the injury and illness statistics.

## Methods

The methods for this systematic review followed the principles outlined in the Preferred Reporting Items for Systematic Reviews and Meta-Analyses Statement (PRISMA) [16]. This study was registered on the International Prospective Register of Ongoing Systematic Reviews (PROSPERO) (Reg No: CRD42019134586) to avoid duplication of the research during the review process.

### Eligibility criteria

Observational, cohort, epidemiological studies assessing the incidence and prevalence of injury and illness in single day and multi-stage mountain marathon cycling races were included in the study, provided they met the criteria of the UCI cross-country marathon category [2]. Prospective and retrospective studies of races longer than 60 km, over one or more days; professional and recreational or amateur events including non-UCI accredited races, of longer than 60 km, and studies that included mountain bikers over 18 years of age were eligible [17]. No date limitation was applied to the studies. Articles written in English or professionally translated into English with evidence of forward and backward translation for accuracy were included.

### Outcome measures

The primary outcome measures were the incidence or prevalence of injury and illness sustained during the event. Secondary outcome measures included the severity and location of the reported injuries, type of injury, rider’s ability to continue in the event, prevalence of injury preceding the event, pre-event training distances and the experience/expertise of the riders (novice, semi-professional, professional). Illness diagnosis, severity and the rider’s ability to continue in the event were additional secondary outcome measures. The definition of injury, illness and severity (of both) used in the study was noted.

### Data sources and search strategy

The following databases were searched: PubMed, CINAHL (EbscoHost), Scopus, PEDRO and the Cochrane library. Congress abstracts from cycling specific and clinical sports congresses (for example Cycling Science Conference and Winter Cycling Congress) held in the past ten years and available online were reviewed to identify unpublished studies. A grey literature search in Google Scholar was performed following the database searches. The reference lists of eligible articles, identified during the search, were manually searched.

Databases were searched using the following keywords: (Mountain OR off-road OR cross-country OR races OR racing OR stage race) AND (Bicycling [MeSH]) OR cycling OR biking OR bikers OR cyclists OR bicycl*) AND (Injuries OR injury OR falling OR Illness OR epidemiology) AND (soft tissue OR fractures OR concussion OR skin abrasions OR gastrointestinal OR respiratory OR dehydration). The database and grey literature searches took place on 5 January 2021 and included all relevant publications up to this date.

Following the keywords search, all abstracts and titles were downloaded to Covidence (Covidence systematic review software, Veritas Health Innovation, Melbourne, Australia. Available at www.covidence.org).

### Data screening and extraction

Two independent researchers (KB and LC) screened the title and abstracts for eligibility based on the above-mentioned criteria. The two reviewers were given independent access to the platform and rated each abstract and title for inclusion or exclusion. Upon disagreement on the inclusion or exclusion of an article, the reviewers discussed and reached consensus on the article’s eligibility.

Following title and abstract screening, both reviewers independently reviewed the full text articles for final eligibility and inclusion into the review. The reviewers discussed and reached consensus on the inclusion of specific studies if there was disagreement between the two reviewers. Once the articles were included, the reviewers extracted the appropriate data from the text.

Data were extracted by the reviewers independently, on participants (age and sex), cycling event (length of stages, total race length, environmental conditions and type of cycling), injury (area of injury, diagnostic practitioner and time off cycling), illness (type of illness, diagnostic practitioner, severity, time off event and whether the rider had a full recovery), study design, and risk of bias using a pre-designed data extraction form.

### Risk of bias and quality assessment

The AXIS tool for critical appraisal of cross-sectional studies was used to assess the reporting quality and risk of bias [18]. The tool provides 20 questions, with seven addressing each quality of reporting and quality of design, and six on potential areas of bias. Each question was answered as ‘yes’, ‘no’ or ‘don’t know/unclear’. The AXIS tool does not provide a numeric scoring system to classify responses as high or low, but allows subjectivity in the interpretation, based on the individual questions [18].

#### Bias assessment

Risk of bias was assessed based on the selection of participants, respondents and non-respondents (and the reason for non-responses) and the internal consistency of the studies. Risk of bias was reported as ‘unclear’ if the required information was not provided by the authors.

#### Quality of reporting

Reporting quality assessment included questions on whether the aims, population and methods were clearly reported in the article. Results and limitations need to be adequately described and discussed. Studies were recorded as ‘unclear reporting quality’ if the content of these questions was not reported in the study.

#### Quality of design

The quality of design was evaluated on its appropriateness for the aims of the study and the justification of the sample size and frame. Conflicts of interests and ethical approval were assessed in this section.

### Data synthesis

Descriptive tables of all data are presented. All injury and illness data were reported as an incidence (per time period) or as prevalence (percentage). Descriptive summary tables were populated with information from each study, including study design, participants, context (events, distance, environment) and outcomes (injury or illness, ability to continue riding).

A narrative analysis of the types, severity and area of injuries and illness type and severity was performed. A quantitative analysis would have been performed if there were three or more studies with sufficient data reported in the same format or with the same definitions of injury and illness, as either incidence or prevalence. Following risk of bias assessment, the same two reviewers decided on the exclusion of the studies in a meta-analysis based on the above criteria. Sub-group analyses of age, sex, type of cycling and experience were not conducted due to the insufficient information available in the studies, and differences in study design and definitions.

## Results

### Search results

**Fig. 1:**
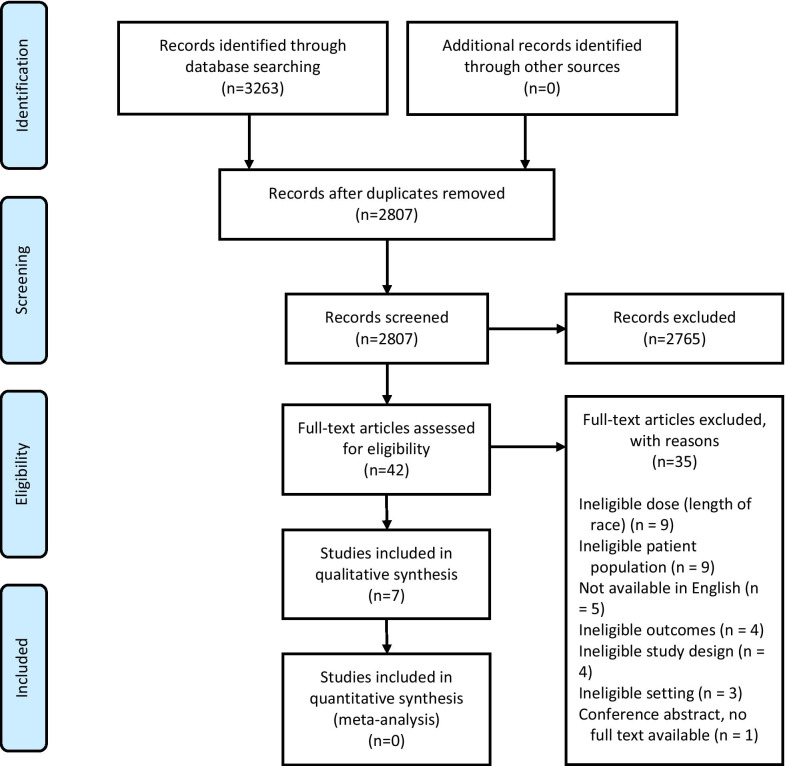
PRISMA flowchart of study inclusions and exclusions

The electronic search returned 3263 references for evaluation (Fig. 1). Forty-two studies were retrieved for full-text evaluation. Seven studies met the eligibility criteria and were included for data extraction and qualitative synthesis. Due to inconsistencies in injury and illness definitions, the studies were not suitable for meta-analysis.

### Characteristics of the studies

Seven studies were included, representing 28,021 participants. Each study had a different design, from retrospective to prospective and surveys, cohort and record audits (Table 1). Four studies reported on injury, with a total of 699 injuries in 15,376 participants [8, 13, 19, 20]. Five studies investigated illness in mountain bike races and reported 1037 cases of illness in 27,634 participants [13, 20–23]. The sex of the participants was not reported in two studies [13, 23], and females were explicitly excluded in one [19]. Two studies used a medical attention definition for both injury and illness [13, 20] and one used the definition of ‘the presence of pain, discomfort or disability’ in a retrospective survey (Table 2) [19]. Lareau and McGinnis [8] did not state their definition of injury.

**Table 1:** General descriptive characteristics of the seven included studies

References	Study design	Number of participants	Race information	Event distance
Griffiths et al. [21]	Retrospective cohort	*n* = 347 (*f* = 45)	Mountain bike event in Wales, UK	Multiple distances: 25–90 km
Lareau and McGinnis [8]	Survey	Cross-country *n* = 111 (*f* = 58); endurance *n* = 337 (*f* = 18)	Mountain bikers taking part in any one of 6 events in the USA	2 × 6-h races 2 × 6–12 h races 2 × 24-h races
McGrath and Yehl [13]	Prospective cohort	*n* = 52	All cyclists in the inaugural Trans-Sylvania Mountain Bike Epic Race	7-day stage race (376 km total)
Mexia et al. [22]	Retrospective cohort	*n* = 11,721 (*f* = 1852)	Birkenbeinerittet race (2009) in Norway	95 km
Stoop et al. [19]	Retrospective cross-sectional observational survey	*n* = 99 (*f* = 0)	Participants of the Swiss Epic Mountain Bike event 2017	5-day stage race (342 km total)
Stuart et al. [23]	Retrospective cohort	*n* = 537	Participants in race in British Columbia	67 km
Taylor and Ranse [20]	Cross-sectional retrospective audit	*n* = 14,777 (*f* = 1847)	All cyclists in the Australian 24-h championships	24-h race in teams (mean of 74 km per cyclist)

*f* female

**Table 2:** Definition and source of diagnosis for injury and illness

References	Definition of injury	Injury diagnosed by	Definition of illness	Illness diagnosed by
Griffiths et al. [21]	–	–	Any gastrointestinal symptoms within two weeks of event	Self-reported
Lareau and McGinnis [8]	Not defined	100% by participant (self-diagnosis)	–	–
McGrath and Yehl [13]	Medical attention	100% by race doctors	Medical attention	100% by race doctors
Mexia et al. [22]	–	–	Diarrhoea within ten days of event	Self-reported
Stoop et al. [19]	Presence of pain, discomfort or disability	100% by race doctors	–	–
Stuart et al. [23]	–	–	More than three loose stools in 24 h between 17 and 26 June 2007 (following race day)	14% laboratory confirmed, remaining 86% self-reported
Taylor and Ranse [20]	Medical attention	100% by race first aid	Medical attention	100% by race first aid

Three studies investigating illness used definitions specifically related to gastrointestinal illness (Table 2) [21–23]. All studies investigated participants of specific races meeting the UCI definition of ‘cross-country marathon’ of 60 km or longer, as per the inclusion criteria. Lareau and McGinnis [8] differentiated between cross-country and endurance races as up to six hours of cycling, and longer than six hours, respectively.

### Quality assessment

The seven studies were assessed on reporting quality, study design quality and risk of bias [18]. The results are presented in Table 3. All studies had an unclear or high risk of bias.

**Table 3:**
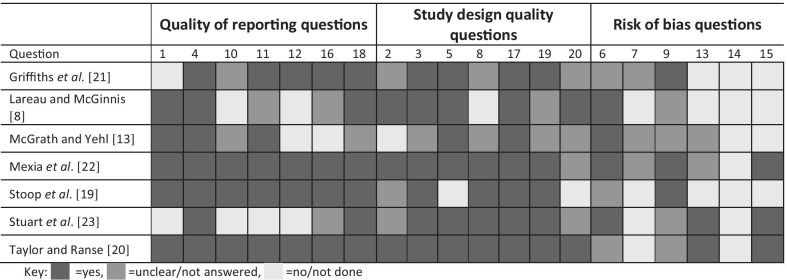
AXIS tool results for reporting and design quality, and risk of bias [18]

### Injury incidence and prevalence

Injury incidence and prevalence are reported in Table 4. There was no standardised definition of injury, injury type or severity of injury across the four studies [8, 13, 19, 20]. Area and type of injury are presented in the table as per the categories reported in each of the studies. The majority of injuries in all studies were skin injuries, including abrasions, contusions and lacerations. Head/neck (concussion) injuries were reported in three studies [8, 19, 20] and soft tissue injuries (including sprains or strains, muscles and ligaments) were reported by all four injury studies [8, 13, 19, 20] (Table 4).

**Table 4:** Injury incidence, type, area, severity and contributing factors in the studies

References	Injury incidence/prevalence	Total number of injuries	Type of injuries *n* (%)	Area of injuries (%)	Severity	Contributing factors
Lareau and McGinnis [8]	Overall: 5.4% Cross-country: 7.2% Endurance: 4.7%	Overall: 25/448 Cross-country: 8/111 Endurance: 17/337	Sprain/strain: 1 (6.7%) Head/Neck (Concussion):2 (13.0%) Skin: 12 (73.3%) Eye: 1 (6.7%)	Head: 7.7% Eye: 2.6% Arm: 15.4% Elbow: 15.4% Wrist/hand/finger/thumb: 12.8% Ribs/trunk/back: 10.2% Gluteus: 2.5% Thigh: 2.6% Calf: 2.6% Knee: 15.4% Shin: 10.3% Skin: 2.6%	Not reported	Not reported
McGrath and Yehl [13]	42.3%	22/52 participants	Bone: 5 (23.0%) Skin: 12 (55.0%) Eye: 1 (4.5%) Soft tissue: 3 (14.0%) Bee sting: 1 (4.5%)	Wrist: 23.0%	Not reported	Not reported
Stoop et al. [19]	Total: 71.0% Amateurs: 68.0% Elites: 74.0%	56/99 participants	Elites Bone: 39.9% Skin and soft tissue: 33.5% Joint: 26.6% Amateurs Bone: 34.1% Skin and soft tissue: 36.6% Head/Neck (Concussion): 4.9% Joint: 24.4%	Elites Knee/calf: 53.5% Ankle/foot: 6.7% Hip/thigh: 13.3% Shoulder: 6.7% Wrist/hand: 20.0% Amateurs Knee/calf: 26.8% Ankle/foot: 4.9% Hip/thigh: 26.8% Shoulder: 26.8% Wrist/hand: 14.6% Head: 7.3% Trunk: 12.2%	Elites Severe: 66.5% Mild: 33.5% Amateurs Severe: 63.4% Mild: 36.6%	Age, exposure time, number of injuries, number of races/year, hours of training/week, protective gear assessed as predictive factors. No significant relationships found
Taylor and Ranse [20]	8.4/1000 h 4.0%	596/14 777 participants Male: 3.8% Female: 5.7%	Bony (fractures): 12 Muscle/ligament: 66 Head/Neck (Concussion): 3 Skin: 359	Limb: 60.0% Head, neck, face: 10.2% Trunk: 2.7% Back: 0.5% Joint: 0.3% Foreign body: 6.5% Eye: 0.9% Multiple injuries: 15.1% Review of injury: 3.7%	Not reported as specific categories 0.5/1000 h were race-ending	Meteorological factors assessed Higher ambient temperature increased risk

Elite: semi-professional and professional cyclists; Amateurs: recreational and amateur; Cross-country: < 6 h; Endurance: 6–24 h of racing, sometimes completed in teams up to four cyclists

### Illness incidence and prevalence

Illness incidence and prevalence are presented in Table 5. Three studies investigated the presence of gastrointestinal symptoms specifically in the days following the event [21–23]. McGrath and Yehl [13] reported that gastrointestinal, respiratory and dehydration symptoms were equal in prevalence (25% each). The final study only reported that headaches and asthma made up 65% of the presenting illnesses with no further information [20].

**Table 5:** Illness incidence, symptoms and ability to continue in the studies

References	Illness incidence/prevalence	Total number of illnesses	Symptoms of illness *n* (%)	Able to continue cycling?	Severity
Griffiths et al. [21]	Total: 46.5% Male: 47.5% Female: 37.8%	161/347 participants	Tiredness: 159 Diarrhoea:151 Abdominal pain: 131 Fever: 94 Nausea: 91 Vomiting: 31 Blood in stool: 15	100% able to continue (symptom onset post-race)	Not reported
McGrath and Yehl [13]	Total: 23.1%	12/52 participants	Gastrointestinal: 3 (25%) Respiratory, asthma: 3 (25%) Neurologic, headache: 3 (25%) Dehydration: 2 (17%) Skin and soft tissue, dermatitis: 1 (8%)	3 withdrawals from race	2 treated in hospital emergency room (1 × gastrointestinal, 1 × dehydration)
Mexia et al. [22]	Total: 4.9%	572/11 721 participants	Self-reported diarrhoea within 10 days of the race: 572 (100%)	100% able to continue (symptom onset post-race)	Not reported
Stuart et al. [23]	Total: 42.0%	225/537 respondents (787 riders in the race)	Cramps: 179 (80%) Fever: 140 (62%) Nausea: 84 (37%) Blood in stool: 29 (13%) Vomiting: 21 (9%)	100% able to continue (symptom onset post-race)	Not reported
Taylor and Ranse [20]	Total: 0.5%	67/14 777 participants	Headache or asthma: 65%	Not reported	Not reported

### Contributing factors to injury

Contributing factors to injury were assessed in two studies [19, 20] (Table 6). Stoop et al. [19] found no significant contributing factors. Taylor and Ranse [20] found that higher ambient temperatures increased the risk for injury.

**Table 6:** Risk factors for illness

References	Risk factors assessed	Significant risk factors
Griffiths et al. [21]	Food and drink from water station Drink from camel pack Other food/drink Ingestion of mud Stayed at camp Drank camp water Ate food from camp Attended pasta party	Other food/drink: RR = 1.36 (*p* = 0.016) Ingestion of mud: RR = 1.79 (*p* < 0.0001)
McGrath and Yehl [13]	Not reported	Not reported
Mexia et al. [22]	Cycling on the second race day Mud in the face or mouth Use of mudguards Taking more than 5 h to finish Cycling ‘on the wheel’ Sex Hydration equipment and fluid type Spitting the first sip of the bottle Use of cycling gloves Participation in previous year Using communal changing area Eating own food Spitting out mud in mouth or rinsing with water Falling	Cycling on the second day: RR = 3.08 (*p* < 0.0001) Mud in the face: RR = 2.85 (*p* < 0.0001) Mud in the mouth: RR = 2.21 (*p* < 0.0001) Not having a rear mudguard: RR = 1.87 (*p* < 0.0001) Not having a front mudguard: RR = 1.83 (*p* < 0.0001) Taking more than 5 h to finish: RR = 1.58 (*p* < 0.0001) Cycling ‘on the wheel’: RR = 1.39 (*p* < 0.0001) Spitting the first sip of the bottle: RR = 1.56 (*p* < 0.0001)
Stuart et al. [23]	Specific food types Use of event hydration facilities Exposure to mud Position and completion of race	Drinking from cups at water station: RR = 1.21 (95% CI 1.44–2.88) Swallowing mud: RR = 2.11 (95% CI 1.50–2.97) Splashed in mouth with mud: RR = 2.05 (95% CI 1.10–3.82) Food contaminated with mud: RR = 1.35 (95% CI 1.05–1.74) Hands covered in mud: RR = 1.82 (95% CI 1.09–3.06) Mud coverage (on body): RR = 1.61 (95% CI 1.10–2.34) Drank water/fluids contaminated with mud: RR = 1.37 (95% CI 1.04–1.81) Finished the race: RR = 3.40 (95% CI 1.36–8.54) Finished in the middle groups (between 4:00 and 6:39 h): RR = 2.63 (95% CI 1.71–4.04)
Taylor and Ranse [20]	Not reported	Not reported

### Contributing factors to illness

In the studies investigating gastrointestinal illness, there were significantly increased risks associated with exposure to mud or ingestion of food and hydration products on the course [21–23] (Table 6). Stuart et al*.* [23] assessed the specific segments in the races where the mud exposure was the greatest and found significantly increased risk in those segments when participants were splashed in the face or swallowed muddy water. The remaining two studies did not assess contributing factors to illness [13, 20].

## Discussion

In this systematic review, we aimed to determine the epidemiology of injury and illness in cross-country marathon mountain biking. No two studies were sufficiently similar in injury or illness definition and reporting, or of sufficiently low risk of bias to allow for meta-analysis [24]. The prevalence of injury ranged from 4 to 71% of participants in the races. The studies with the lowest prevalence of injury of 4–5% were both investigating single day races ranging from under 6–24-h races (in teams) [8, 20]. The prevalence of injury in the multi-day stage races (5–7 days) ranged from 42 to 71% [13, 19]. The difference in prevalence could be attributed to the access to medical assistance in multi-day stage races and the need for the cyclists to be attended to before the next day of racing. Cyclists who have minor injuries like skin abrasions or lacerations during multi-stage races may make use of the medical services to clean and dress wounds before riding the following day. It is possible that these same injuries in a single-day race may be managed by the cyclists themselves once they return home.

The use of standardised definitions of injury and illness in epidemiological research is essential to enable comparisons between studies and develop a clear understanding of the burden of both injury and illness [7]. Injuries should be anatomically classified according to the Orchard Sports Injury Classification System (OSICS) with additional information on the location of the injury on the event course, onset of injury, mechanism of injury and contributing factors collected [25, 26]. Information on the non-reported medical problems could be gathered via self-report questionnaires or interviews with participants to provide a more complete analysis of the event [7].

Medical attention injury definitions were used in two studies (50%) [13, 20], defining injury as only those who requested assistance from medical staff. The risk of using medical attention as an injury definition is that minor injuries that cyclists may be able to manage themselves, or overuse injuries that are not severe enough to prevent riding, would be missed. One study [8] did not report their definition of injury, and can therefore not be replicated, or compared to other studies. Use of standardised classifications will allow for future studies to be compared and combined for analysis.

The majority of injuries reported in these studies were skin-related [8, 13, 19, 20]. Blisters were classified as a repetitive or gradual onset injury, while lacerations, abrasions and contusions would be considered sudden/acute injuries [27]. Concussions were reported in three of the four studies, self-diagnosed in one and diagnosed by medical professionals in the other two (Table 2) [8, 19, 20]. An additional study, not meeting the inclusion criteria for this review, has investigated the incidence of concussion in cross-country marathon cyclists [28]. Concussion can have potentially severe long-term sequelae and needs rapid assessment and withdrawal from training and competition [29]. In all three studies in our review reporting concussion [8, 19, 20], it was not cited as a race-ending injury. This could be due to delayed onset of symptoms or a lack of awareness and assessment of concussion by cyclists themselves [28]. Clark et al. [28] found that of 40 cyclists who reported at least one symptom of concussion over the previous year, only 12 were medically diagnosed with concussion. Sixty-eight percent of cyclists continued to train and compete while experiencing symptoms of concussion [28]. It is unclear whether these cyclists did not have sufficient knowledge to recognise these symptoms, and the dangers of participating while concussed, or whether they wilfully continued to ride in spite of the concussion. Further research in this area is needed to understand the mechanisms behind this behaviour.

The severity of injuries was only reported in a single study on the 2017 Swiss Epic event [19]. Severity of medical encounters has been described by Schwellnus et al. [25] as *minor* (no withdrawal from event required), *moderate* (withdrawal from the event, with non-life-threatening injuries) and *serious/life-threatening* (transport to hospital/referral to high level of care). There are additional categories for event-related sudden cardiac arrest, sudden cardiac death and sudden death [25]. These differences in definitions make it difficult to compare studies, and also to determine the true burden of the injuries experienced in these events.

Only two studies assessed factors affecting injury [19, 20]. None of these factors investigated by Stoop et al. [19] were found to be predictive of injury in this specific race for either elite or amateur cyclists. Taylor and Ranse [20] found that ambient temperatures were associated with injury rates. Higher temperatures, over 20 °C, were significantly related to greater number of injuries [20]. The authors surmised that higher temperatures may increase fatigue and dehydration, but acknowledged that other factors may also contribute to this relationship [20]. The effect of progressive fatigue over multi-stage races and the distribution of injuries across the stages are additional factors that should be considered, together with the effect of temperature on fatigue and injury.

Differences between race settings (including terrain and temperature), length of race, availability of onsite care, and experience of cyclists may contribute to the vastly different findings between the studies. Over the 10-year period of the included studies, cycling technology has also resulted in equipment changes to bicycles, shoes, helmets and clothing. The protective effects of this equipment specifically within this category of cycling remain unknown. Without regular and ongoing epidemiological data collection using standardised injury definitions, the true burden of injuries will remain uncertain.

Three of the studies investigating illness exclusively assessed gastrointestinal-related illnesses [21–23]. Other illness conditions were not investigated in these studies. One study [20] reported illness prevalence, but the primary objective was to assess injury, and therefore detail of the illnesses is lacking. Both Griffiths et al*.* [21] and Stuart et al. [23] conducted their studies as a response to reports of gastrointestinal illness following the races, while the study by Mexia et al*.* [22] aimed to assess whether preventative measures against gastrointestinal illnesses were effective in reducing the incidence of such illnesses. While the investigation of illness onset post-event will assist with the development of prevention strategies for these gastrointestinal illnesses specifically, it does not allow for adequate preparation of medical facilities provided during the race.

The onset of illnesses in mountain biking may be delayed due to the incubation nature of some viral and bacterial infections [7]. Symptoms related to these illnesses may only appear in the days following the event, as in the case of gastrointestinal illness. While these illnesses are not managed during the race by the medical team and therefore do not contribute directly to the race burden, knowledge of these illnesses presenting in the period following the event is still essential to allow race organisers to implement possible preventative measures. In multi-day stage races, it is possible that gastrointestinal diseases will require medical care if contracted within the first days of the event. Therefore, ongoing monitoring of participants after the event would provide helpful insight into these illnesses.

## Summary and conclusion

All studies included in our review had different study designs and injury/illness definitions while purporting to have the same goal. The findings of this systematic review show that further epidemiological studies are needed in cross-country mountain biking, with standardised injury and illness definitions and recording methods. There is sufficient evidence that injury in mountain biking is an area of concern, but consistent reporting will allow for greater comparison between studies and a better global view of the injury burden. The epidemiology of illness in cross-country mountain biking is unclear and warrants further investigation. Epidemiological data are essential for risk assessment and understanding injury with a view to reducing injury and illness in these events.

## Data Availability

Not applicable.
